# Supplementary material for: Individual differences in bottom-up and top-down emotion generation
Source: PLOS Ment Health. 2026 Jan 16;3(1):e0000452. doi: 10.1371/journal.pmen.0000452 (PMC12810820; doi:10.1371/journal.pmen.0000452)
Supplement: S1 Table — Correlation strengths are defined as: weak r = .1-3, moderate r = .3-.5, strong r = .5-.8. (DOCX) [file pmen.0000452.s001.docx]

S1 Table

*Predicted Correlations Between the New and Extant Measures*

*Primary and Secondary Measures of Interest and their Relation to Bottom-Up and Top-Down Emotions*

| Primary Measure | Predicted Correlation | Related Process | Description |
| --- | --- | --- | --- |
| Emotion Reactivity Scale | Top-Down and Bottom-Up, *r* =.3-.5 | Both | - Measures emotional reactivity - A total score and three subscales; sensitivity, intensity, and persistence - Magnitude and duration of emotional response. |
| Dimensions of Openness to Emotions | Top-down and Bottom-up contingent upon sub-scales, *r* =.3-.5. | Both | - Measures components of emotion processing. - Five subscales are measured: Perception of Internal Bodily Indicators of Emotion, Perception of External Bodily Indicators of Emotions, Regulation of Emotions, Communication and Expression of Emotions, and Cognitive Conceptual Representation of Emotions. Assesses for bodily engagement and self-awareness and identification of an emotional response. |
| Cognitive Mediated Beliefs Questionnaire | Stimulus-Response:  Bottom-Up, r =.3-.8. Cognitive Mediation:  Top-Down, *r* = .3-.8 | Both | - Measures the extent to which individuals believe that emotions are either cognitively mediated or that emotions occur through an external stimulus-response process - Two sub-scales: Cognitive Mediation and Stimulus-Response |
| Need for Cognition Scale | Top down, r =.3-.8 | Top-Down | - Tendency and preference to engage in thinking. |
| Rumination-Reflection Questionnaire | Reflection:  Top-down, r =.3-.5  Rumination:  Top down, *r* =.3.5 Bottom-Up, *r* =.3-.8 | Top-down | - Tendency to engage in reflective and recurrent thoughts. - Two sub-scales: Reflection and Rumination. |
| Cognitive Flexibility Inventory | Top-down, .3-.8 | Top-Down | - Measures the tendency of to replace maladaptive thinking with more adaptive thinking. - Two sub-scales: Control and Alternative - A single cognitive flexibility factor is measured. Assesses for the need for cognition during an emotional response. |
| Highly Sensitive Person Scale | Bottom-up, r =.3-.8 | Bottom-Up | - Measures sensitivity to sensory stimuli. |
| *Emotion Regulation Questionnaire* | *Suppression:*  *Bottom-up, r = -.3 to .5*  *Reappraisal:*  *Top down, r = -.3 to .5* | *Both* | - *Measures emotion regulation* - *Two sub-scales: Expressive Suppression and Cognitive Reappraisal.* |
| *Beck Depression Inventory* | *Top-down, r = .1-.3* | *Top-down* | - *Self-report symptoms of depression.* |
| *State-Trait Anxiety Inventory* | *Bottom-up and Top-down, r = .1-.3* | *Both* | - *Self-report symptoms of anxiety for both state and trait-level* |
| *Big-Five Inventory* | *Neuroticism:*  *Top-down, r = .1-.3* | *Top-down* | - *Assesses personality indicator: Extraversion, Openness, Conscientious, Neuroticism and Agreeableness.* |

*Note*. Correlation strengths are defined as: weak *r* = .1-3, moderate *r* = .3-.5, strong *r* = .5-.8
