# Supplementary material for: Individual differences in bottom-up and top-down emotion generation
Source: PLOS Ment Health. 2026 Jan 16;3(1):e0000452. doi: 10.1371/journal.pmen.0000452 (PMC12810820; doi:10.1371/journal.pmen.0000452)
Supplement: S2 Table — (DOCX) [file pmen.0000452.s002.docx]

S2 Table

*Factor Loadings of the CFA*

| Item | Standardized Factor Loading | α | Factor correlation |
| --- | --- | --- | --- |
| Factor 1: Top-down |  | .80 | .535 |
| My thoughts tend to cause some sort of emotional response in me. | .807 |  |  |
| When I’m feeling emotional, it’s often because of thoughts I’ve had. | .644 |  |  |
| When I am feeling emotional it’s often because of how I have interpreted a situation. | .543 |  |  |
| My thoughts often affect how I feel. | .579 |  |  |
| How I interpret conversations tends to affect my emotions. | .639 |  |  |
| When I experience negative emotions, I tend to get caught up in my thoughts. | .716 |  |  |
| Factor 2: Bottom-up |  | .57 | .535 |
| Viewing emotional images tend to cause an emotional response in me. | .575 |  |  |
| My emotions often arise from experiencing things in my environment (e.g., sounds, images, or scenes). | .395 |  |  |
| My emotions come from physical properties of a situation such as the scenery and sounds of a location. | .239 |  |  |
| I tend to react to displays of emotions by others. | .707 |  |  |
