# Supplementary material for: Individual differences in bottom-up and top-down emotion generation
Source: PLOS Ment Health. 2026 Jan 16;3(1):e0000452. doi: 10.1371/journal.pmen.0000452 (PMC12810820; doi:10.1371/journal.pmen.0000452)
Supplement: S3 Table — (DOCX) [file pmen.0000452.s003.docx]

*Exploratory Analysis: Comparison to the CMBQ*

The hypotheses for this study were also examined for the CMBQ measure given that it potentially measures similar underlying constructs. In contrast to the bottom-up top-down measure, the CMBQ’s C-M scale was positively associated with measures of cognition including the Reflection subscale of the RRQ (*r*(223) =.3, *p <* .001), both CFI subscales (*rs*(223) =.34, .26, *p* < .001 , the BIF (*r*(223) = .320, *p* = .002), and the NCS (*r*(223) = .25, *p* < .001). Notably, the C-M subscale was not associated with the top-down factor, suggesting the two scales were not related as hypothesized. The S-R sub-scale was positively associated with the bottom-up factor (*r*(190) = .24, *p* < .001) and the HSPS (*r*(223) = .27, *p* < .001), consistent with predicted outcomes. It was also positively associated with the top-down factor (*r*(190) = .36, *p* < .001).

For discriminant validity, the C-M scale did not correlate with any of the external stimuli measures, nor was it associated with the S-R scale, suggesting they are measuring distinct constructs. In addition, the S-R subscale was negatively correlated with certain measures of cognition including the CFI Control subscale (*r*(223) = -.34, *p*  <.001), and the NCS (*r*(223) = -.19, *p* = .005), further supporting selectivity of the sub-scales. Lastly, the C-M sub-scale was positively associated with the Reappraisal subscale of the ERQ (*r*(223) = .56, *p* < .001), was negatively associated with STAI-Trait (*r*(223) = -.19, *p* = .004) and Neuroticism (*r*(223) = -.19, *p* = .004). In contrast, the S-R sub-scale was positively associated with the BDI-II (*r*(223) = .22, *p* = .001), STAI-State (*r*(223) = .23, *p* < .001) and STAI-Trait (*r*(223) = .3, *p* <.001), and neuroticism (*r*(223) = .32, *p* < .001; see Table 8 for a comparison of the CMBQ and bottom-up top-down measure). Overall, the correlation results from the CMBQ better aligned with the initial predicted hypotheses than the bottom-up top-down measure.

S3 Table

*Comparison of the CMBQ and BUTD Measure*

|  | Top-Down | Bottom-Up |
| --- | --- | --- |
| Top-down Factor | – | .66* |
| RRQ - Rumination | .44* | .26* |
| RRQ - Reflection | -- | -- |
| CMBQ - C-M | -- | -- |
| CFI - Control | -.25* | -- |
| CFI - Alternative | -- | -- |
| DOE - Communication and Expression of Emotions | -- | .16* |
| DOE - Cognitive Conceptual Representation of Emotions | -- | -- |
| NCS | -.15* | -- |
| BIF | -- | -- |
| ERS | .55* | .38* |
| BU Factor | .66* | – |
| HSPS | .51* | .38* |
| CMBQ-S-R | .36* | .24* |
| DOE - Perception of External Bodily Indicators of Emotion | .18* | .21* |
| DOE - Perception of Internal Bodily Indicators of Emotion | .29* | .28* |
| Big-Five Inventory - Neuroticism | .41* | .22* |
| ERQ - Reappraisal | -- | -- |
| ERQ - Suppression | -- | -.15* |
| DOE - Regulation of Emotions | -.41* | -.33* |
| BDI-II | .37* | .22* |
| STAI - Trait | .32* | .23* |
| STAI - State | .32* | .23* |
