# Supplementary material for: Individual differences in bottom-up and top-down emotion generation
Source: PLOS Ment Health. 2026 Jan 16;3(1):e0000452. doi: 10.1371/journal.pmen.0000452 (PMC12810820; doi:10.1371/journal.pmen.0000452)
Supplement: Attachment — Submitted filename: BUTDIDPMEN_RR_clean (1).docx [file pmen.0000452.s005.docx]

*Dear Dr. Randall Waechter,*

*We were grateful to receive the comments from reviewers and have read through them carefully. We have made several changes to our manuscript based on the reviewers’ feedback which we believe have substantially strengthened the manuscript. Please see our responses to reviewer’s below in italics.*

*In addition, we have updated the submission based on requests from the original email notifying us of the decision for a revision including: An updated Financial Disclosure statement, Data Availability Statement ,separate files for figures, an updated reference lists, and removed our supporting information from the main submission. Please do not hesitate to reach out should other requests arise.*

Reviewer #1:

R1.1.This is well-written and clear.

*We appreciate the reviewer’s positive feedback and hope they enjoyed reviewing the manuscript.*

R1.2.Abstract doesn't contain any numeric detail (sample size, results)

*We have updated our abstract to include sample size of each study and several key results.*

R1.3.Introduction seems to neglect personality traits - not true that 'less has been done to capture individual variation'. There is a large literature on personality traits, individual differences and emotion.

*We agree there is a rich literature on personality traits, individual differences, and emotion. We have changed this language (here and elsewhere) to acknowledge this established literature. We hope this highlights the critical gap our paper intended to address, individual differences in bottom-up and top-down emotion generation specifically.*

R1.4.Structure is odd - far too early to sat 'We sought to determine' - research questions should go at the end of an introduction. Later, again the suggestion is made that 'less emphasis has been put on the importance of emotion generation'. There is a vast literature on this already. There should be a 'Methods' section containing Study 1/2/3, rather than 'Study 1' being the first heading.

*Thank you for this - we have moved our research questions to the end of the introduction.*

R1.5. It is surprising to use Prolific for Study 1, then a student sample for the confirmatory part. I would have done this the other way around, so that the general population sample is the validation sample. What is the rationale for this?

*We agree that it is best to validate a measure with a more general sample when possible. Due to funding constraints at the time the study was conducted, we were unable to utilize a platform such as Prolific for a more generalizable sample. However, we were able to use Prolific for our third study and ran the CFA again, so ultimately, we were able to use a general sample as a validation sample.*

R1.6.Table 6 - at first glance I thought this was a one-factor model due to layout. Can you spread across two columns for clarity.

*We have modified the table by adding a column to better visually represent the two-factor structure (Lines: 9545-9552).*

R1.7.Table 7 - despite the two-factor structure, the pattern of correlations with validating scales suggests a general factor of negative emotionality.

*We agree, the pattern does indicate a high level of overlap between both BUTD subscales and established measures of negative emotionality. However, we found not all established measures were correlated with both subscales. Furthermore, we saw some evidence that certain measures might be correlated more strongly with one factor than the other, suggesting possible distinction. In particular, there is some indication that the top-down subscale may be especially related to cognitive aspects of emotional reactivity. We highlight this in the new manuscript (results and discussion, including limitations) on page 30, 40, and 47. Additionally, we rigorously tested the best fit of the data several times across independent datasets and found the two-factor structure consistently provided a better fit, suggesting these are still somewhat separable constructs. This pattern of results warrants more follow up to further clarify the utility of the measure and its two separate subscales. Ultimately, we hope that readers of this manuscript conclude that this new measure has a two-factor structure and that more work is needed to identify exactly how those factors may differ.*

R1.8.Study 3 - this was not pre-registered but there was a clear research question. Why couldn't that be pre-registered?

*To align with best practices in the field, we agree that preregistration should be used when possible. Across our three studies, Study 1 and 2 had clear exploratory and confirmatory goals, respectively. To reflect these goals, we preregistered Study 2. While we did have clear questions we intended to answer in Study 3 (e.g., does our measure correlate with an affective task), we didn’t have strong predictions about how the task and measures would relate. Thus, Study 3 was exploratory in nature. We now caution in the limitation section that correlations with the task in study three should be considered preliminary and replicated in future studies (10140-10141). In addition, Study 3 was the result of additional funding associated with a separate research project with time-limited access to the funds. We thus took the opportunity to explore this relationship between task and self-report data without preregistering to ensure we could collect the data before the funding expired.*

R1.9.Proofreading is needed throughout - several references are missing square brackets and the number is not formatted correctly in the text. L849 has "=" for example. Missing reference at L882.

*Thanks for catching these small errors. We have carefully proofread the manuscript and made corrections as needed.*

R1.10.Was there an opportunity to record reaction time during Study 3 tasks? Might reaction time offer insights into speed of processing of top down / bottom up emotion generation?

Does speed of processing of information in the environment relevant to emotion impact emotion, so it becomes a cognitive skill of sorts? Is there a possible evolved mechanisms for this that increases survival e.g. threat perception being accurate and fast?

*We agree reaction time may provide insight into the processing of differently generated emotions. Unfortunately, we did not record reaction time in Study 3. We think this is an excellent question to explore in future studies. As a result, we have added this as a future direction (Lines: 10246-10250).*

R1.11.The discussion section should be rewritten to consider more closely strengths and weaknesses in relation to existing studies, to address concerns that these constructs are jangles for existing constructs including neuroticism. Limitations of self vs informant reports need acknowledging.

*We thank the reviewer for this recommendation. During the conception of this project, we drew on well-established theories in the emotion literature, and established measures of emotional responding (including the emotion regulation questionnaire and the cognitive mediated beliefs questionnaire) to help motivate our item development and validity instruments. As a result, we report on many of the same questionnaires and constructs these established measures used to assess our measure. Mirroring the methods and instruments used in these previously established measures increases our confidence that our measure has been appropriately assessed in the context of emotion theory. However, there were several we did not include.*

*While we did see moderate correlations with neuroticism in Study 2, the strength of the correlations was not strong enough to suggest that the BUTD measure is simply a jangle. In addition, as we mentioned above, the two factors, while related, appear to be distinct such that the top-down scale appears to be measuring cognitively generated reactivity specifically. We believe the BUTD measure contributes novel understandings to the emotional reactivity literature, namely how cognitively generated emotion may contribute to emotional reactivity. We have adjusted our conclusion to reflect these strengths and weaknesses as well as the utility of considering peer/other reporting of BUTD emotional responding in future work (Lines:10094-10115 ;10122;10130-10142.*

R1.12.There is some discussion of implications for understanding psychopathology, but the scales used to validate your constructs focused on normal rather than abnormal constructs. At L788 - without stepping too far beyond the data, what are the possible implications?

*We appreciate this guidance on trying to balance potential implications with staying close to the data. We did include commonly used measures of psychopathology that indicated associations with the BUTD measure. Given that our results suggest higher TD scores are associated with more negative emotions (and less positive emotions), this could result in increased risk for psychopathology given negative emotionality is a core feature of many mood and anxiety disorders.*

*We also used established measures that have demonstrated relationships with psychopathology. For example, rumination, emotion reactivity and neuroticism are associated with psychopathology. Rumination in particular is a common symptom of depression. Related, we included measures that have also been shown to be negatively correlated with psychopathology, such as cognitive flexibility. While these are not direct measures of psychopathology in and of themselves, they are often considered risk or protective factors for psychopathology. We do not believe our BUTD measure is a direct measure of psychopathology, but rather that variability in BUTD generated emotions may possibly be related to its development (e.g., heightened reactivity to cognitively generated emotions may result in higher levels of maladaptive thinking patterns, which are thought to be at the core of emotional disorders such as depression), and/or its maintenance (without active intervention challenging well-established thinking patterns, the reactivity is likely to persist).*

R1.13.Avoid "it's" throughout, which sounds informal ("it is").

*Thank you – we’ve replaced it’s with “it is” throughout.*

R1.14.Add to limitations, predictive validity for (longitudinally measured) outcomes lacking (e.g. health outcomes). Relationship to internalising/externalising psychopathology needs further study, and the "p" factor.

*These are excellent suggestions for future study. We have added these to our limitations section as suggested (Lines: 110157-10159).*

Reviewer #2:

R2.1This paper has been written fairly well. However, I would like to suggest a few points that would make the paper more eligible for publication.

There is no scientific justification for the need of this research and what is the added value of this findings to the medical/scientific fraternity.

*We appreciate the invitation to add more of our rationale for conducting the study. Previous literature on bottom-up and top-down emotion generation has provided meaningful insights into key brain mechanisms involved in emotion generation. It also illustrated how these differently generated emotions interact with commonly studied adaptive emotion regulation strategies. For example, research has shown that using a top-down based emotion regulation strategy in the context of top-down generated emotions appears to improve regulation success. By comparison, this same strategy may be less effective for bottom-up generated emotions (see McRae et al.,2012 cited in our manuscript for more details). Creating a self-report measure that captures this variability would allow for a more efficient and less invasive method for measuring these constructs.*

*A 10-item measure that may have direct implications for teaching individuals strategies to regulate emotions has high potential for clinical utility with low burden to both the individual seeking treatment and the clinician. Research indicates individuals with anxiety may more readily perceive ambiguous stimuli as threatening. It’s possible that BU and TD variability in emotion generation may contribute to this bias, given that individuals who score more highly on the BU scale may be more likely to demonstrate heighted awareness to perceptual stimuli in the environment. Given we found that the BUTD measure was related to psychopathology as hypothesized, the BUTD measure might also help identify specific treatment targets for intervention and emotion regulation strategies most likely to be effective given an individual’s unique endorsement of bottom-up and top-down emotions. Considering the above example, clinically this may indicate that a behavioral approach that targets reactivity to those environmental stimuli (versus a cognitive approach) could be more successful in reducing symptoms. We have updated our Clinical Relevance section in the Discussion to reflect the utility of assessing for bottom-up and top-down generated emotions in the context of treating anxiety and depression (Lines 9962-1006)*

R2.2.It is imperative to include the location of research, sample selection criteria, inclusion and exclusion criteria and method of data collection.

*Thank you for pointing this out -- we have made several changes to our manuscript to ensure these study and sample characteristics are clear across all three studies (Lines: 9648).*

R2.3.The discussion section should include more studies that support or refute your findings. More references are needed from the recent studies conducted.

*Thank you for inviting us to expand the connection to the recent literature. We have added citations from recent or relevant papers throughout the discussion (Lines: 9846;9899;10001-10002)*

Reviewer #3:

R3.1.This is an interesting and important set of data that has implications for the treatment of emotion processing disorders. Whilst the validation of the data has been conducted with relative rigour the context of the research question could benefit from a more detailed theoretical framework.
It would benefit from a more nuanced and detailed examination of the foundations for the research question.
I would like to note that I enjoyed reviewing this manuscript.

*We are delighted to hear this reviewer enjoyed reviewing our manuscript and have addressed their more specific recommendations below. We are hopeful the reviewer will be satisfied with our responses and the changes we made to the manuscript.*

Introduction
R3.2.Whilst there are supporting citations regrading “top-down” and “bottom-up” it might be helpful to add a little more context. Especially as the terms originated in work in visual perception in the late ‘60’s and into the 1970’s. I am not suggesting that these origins need to be detailed just perhaps a little more context in terms of the exogenous and endogenous aspects to clarify the terms in the context of emotion. Whilst this is expanded upon later in the introduction, I am not convinced that this adequately address the complexity of the relationship between a stimulus driven response and a cognitive or biologically driven response. The relationship between the two is perhaps oversimplified here?

*We agree with the reviewer that there is a complex history of these two terms. Describing top-down to be more “endogenous” and bottom-up to be more “exogenous” is yet another way to consider these dynamics. To the reviewer's point, the affective science literature drew on visual processing literature that considered bottom-up processing to be from sensory based stimuli, versus top-down which is more so influenced by context and appraisals based on knowledge. We have added additional context earlier in our introduction to better define and underscore the complex relationship between each type of emotion (Lines: 110-128;209-223).*

R3.3.Lines 50.51: I am not sure that its clear what is meant here in relation to individual differences and group differences in relation to emotion in relation to the above. This could be expanded and made more explicit.

*We have tried to expand this section to make it clearer that we are highlighting fewer research efforts have been to identify individual variation in bottom-up and top-down emotions ,especially in the context of self-report measures (Lines:56-62).*

R3.4.Lines 83-96:
I think its important to distinguish between the structural accounts of emotion processing in relation to TD-BU and the temporal accounts in imaging studies in ERP/EEG studies which might help shed some light on these concepts.

*We share your interest in aligning the bottom-up and top-down distinction with temporal accounts of neural processing of emotional information. Namely, discerning whether bottom-up and top-down generated emotions are qualitatively different types of generation, or more reflective of a “slower” versus “faster” form of emotion generation remains opaque. We have added a section in our future directions that reflects the importance of including temporal accounts and neuroimaging in future studies to begin to address this (Lines: 18976; 19170-19175;19275-19277).*

R3.5.The section on psychopathology would benefit from a more detailed account. There is actually quite a large literature on these issues that would support a stronger and more balanced argument about how endogenous and exogenous accounts relate to TD BU accounts in the psychopathology of emotion processing disorders.

*We have provided additional context and references in our discussion to reflect the literature on endogenous and exogenous accounts of emotion processing (Lines: 10001-10006). We agree that there is a large literature on distinct and overlapping processing across internalizing disorders. Considering the impact of bottom-up versus top-down emotions in contributing to these distinctions and overlap may provide additional insight.*

Methods
R3.5.Line 184: This is a good example of where the explicit foundations for the research question are implicit in the supporting literature raised in the introduction. I would suggest working backwards to expand support for this claim in a more detailed examination of the literature that supports this claim.

*Thank you for providing us with this example to further contextualize your recommendation. We believe the additions we have made to the introduction address this specific concern.*

R3.6.The co-production aspect of the study is a strong positive point.

*We agree the interdisciplinary nature of our author team is a strength of the project, thank you.*

R3.7.Line 210: I am assuming this is a typo? Dat/Data?

*Thank you for noting this, we have fixed this typo.*

R3.8.I would argue there needs to be a stronger argument for use of the Beck over other measures like the CES-D.

*We appreciate your raising the important issue that we used different assessments for depression symptoms. Both the BDI and CES-D are comprehensive measures of depressive symptoms with relative strengths and weaknesses. For example, both have demonstrated strong reliability and validity for decades. While the CES-D can be helpful in detecting depressive symptoms in a broader sample, the BDI is often used to more comprehensively assess depression symptoms in a clinical setting. Given our interest in determining whether the BUTD measure may have clinical application (as opposed to general population detection of depression), we believe the BDI is more appropriate for the purposes of our research. However, given we did not specify a clinical sample in our studies, including the CES-D in future work may be advantageous, especially for non-clinical samples. It should also be noted that the CES-D and BDI are strongly positively correlated across various samples. We have recommended the exploration of depression symptoms using a measure like the CES-D in our discussion section (Lines 19022-19035).*

Jiang, L., Wang, Y., Zhang, Y., Li, R., Wu, H., Li, C., Wu, Y., & Tao, Q. (2019). The Reliability and Validity of the Center for Epidemiologic Studies Depression Scale (CES-D) for Chinese University Students. *Frontiers in Psychiatry*, *10*. <https://doi.org/10.3389/fpsyt.2019.00315>

Santor, D. A., Zuroff, D. C., Ramsay, J. O., Cervantes, P., & Palacios, J. (1995). Examining scale discriminability in the BDI and CES-D as a function of depressive severity. *Psychological Assessment, 7*(2), 131–139. [https://doi.org/10.1037/1040-3590.7.2.131](https://psycnet.apa.org/doi/10.1037/1040-3590.7.2.131)

Wilcox, H., Field, T., Prodromidis, M., & Scafidi, F. (1998). Correlations between the BDI and CES-D in a sample of adolescent mothers. *Adolescence*, *33*(131), 565–574.

R3.9.It might be helpful to discuss the role of self-report measures in the context of emotion processing.

*Thank you for this – we’ve added a discussion of the limitations of self-report to the manuscript (Lines: 19173-19175). As with many methods in psychology there are relative strengths and weaknesses of a given method of choice. We were motivated to move away from expensive objective measures (fMRI) toward self-report measures for time, accessibility, and feasibility purposes. However, self-report measures have their own biases. For emotion, they're dependent upon access to/awareness of one's own emotions.*

R3.10.Was there support offered for those participants who scored highly on the Beck for example? Was there any data collected in regard to participants who might be prescribed anti-depressants? Or have a history of being prescribed as research is clearly supporting an extremely long withdrawal in terms of years rather than weeks to SSRI’s in particular? Some data suggests levels at 60% in undergraduate populations.

*We appreciate the reviewer’s attention to the wellbeing of our participants. All three studies included a risk protocol. The online survey platform was programmed to send an email notification to the study team if someone endorsed any level of suicidality on the Beck Depression Inventory or PHQ-9. When this occurred, participants were provided with a resources page that included a variety of nationwide and local mental health resources including crisis lines. In addition, participants who were enrolled at the authors’ home institution were provided with follow up that aligned with the institution’s guidelines for supporting students with mental health concerns (e.g., risk assessment by study staff, and/or trained student support staff outreached the student). While we did not formally ask participants to disclose their use of anti-depressants, some participants spontaneously self-disclosed their use of such medications in a free response at the end of the survey. We have added a section to our discussions that suggests future work should specifically request this information and analyzes this data in relation to the BUTD measure as relevant (Lines: 192129-19131)*

R3.11. Was the criteria for participation only age? Was there any consideration of other demographic and psychological variables? Emotion processing disorders, screened or self-reported for example?

*Our primary criteria were age (between 18-55), and current enrollment in a United States college or university (this was only true for Study 1, and a portion of Study 3). We did not screen for emotion processing disorders, as we were interested in variation across clinical and nonclinical samples.*

Results
R3.12.The treatment of data is reported well. However there are some interesting interpretations that need unpacking and again, perhaps highlight some of the oversimplistic theoretical framework that drives the research. For example the introduction of the role of threat accounting for BU responses.

*The reviewer highlights an important point regarding the inherent questions the data are attempting to answer. Are bottom-up and top-down generated emotions differentiated by mechanistically different responses in the brain, or by speed of processing? In other words, are cognitive versus perceptual processes actually different? It could be simply that bottom-up tends to be faster or happen first in sequence given that external sources of emotion may pose a physical threat that warrant faster response time from an evolutionary perspective. Whereas top-down is “slower” given it’s less grounded in threat detection. Based on the available literature highlighting distinct neural mechanisms, we believe that while there are some overlapping processes (e.g., both often elicit amygdala activation), they appear to be separable processes given that the top-down generation of emotion involves prefrontal regions that are not observed during generation of bottom-up emotions. Thus, our interpretation of our results is grounded in that theoretical background. We hope our additions to the introduction and discussion as well as response to your comments above further clarify our interpretations of results.*

R3.13.Line 511: It would be interesting to see if this was any different using a measure of Cognitive fusion rather than psychological flexibility? I would be interesting to examine this in light of ACT therapy.

*We agree it would be interesting to examine the measures’ relationship to psychological flexibility. We have added this to our future directions section of the discussion (Line 19195).*

R3.14. Line 623: there seems to be an error in citation here?

*Thank you for noting this. We have corrected the error.*

R3.15.PQH-9 & GAD-7 as diagnostic measures are very different in interpretation that the Beck and STAI for example? Maybe this should be expanded on in more detail?

*Thank you for this suggestion and highlighting the nuance of interpretation across these various measures. We have expanded on the differences between these measures, and how they should be interpreted in relation to one another and the BUTD measure in the discussion (Lines: 19137-19156).*

Discussion
R3.16. In the general discussion the results presented in relation to structural accounts of emotion processing are a little tenuous. I would temper those. The measure developed in relevant and interesting but to make those claims support from imaging data would be necessary.

*We appreciate the reviewer’s attention to ensuring we are accurately interpreting results. We have reviewed our discussion and made modifications to ensure we are appropriately couching our interpretations in the context of the broader literature. We have also made additional suggestions for how to further validate the measure (e.g., assessing its predictive validity, validating it against other objective measures of emotion processing) to ensure the suggestions we make about its utility are supported by data (Lines: 18928; 18931;18938-18942; 18947-18949).*

R3.17.Whilst the authors note the limitations of their sampling there are other limitations that might make interpreting the data difficult. More detailed information in regard to the sample would be helpful? Recreational drug use, prescribing history etc.

*We agree it is always helpful to have comprehensive information on the study sample. We did not require participants to disclose this information, although a portion of them did spontaneously (see response above). These data will be available to review once the paper is published should others be interested in examining this information in the subsample that provided it. We have also added this as a future direction (Lines: 19250-19252).*
